# Notch NICD domains form biomolecular condensates

**DOI:** 10.1002/ccs3.70039

**Published:** 2025-07-26

**Authors:** Tana R. Gazdik, Anamaria Zavala, Henry A. Charlier, Michael A. Detweiler, Jacob J. Crow, Tyler Lawton, David Oke, Saborni Sarker, Allan R. Albig

**Affiliations:** ^1^ Biomolecular Sciences PhD Program Boise State University Boise Idaho USA; ^2^ Department of Mechanical and Biomedical Engineering Boise Idaho USA; ^3^ Department of Chemistry and Biochemistry Boise State University Boise Idaho USA; ^4^ Biomolecular Sciences Master's Program Boise State University Boise Idaho USA; ^5^ Department of Biological Sciences Boise State University Boise Idaho USA

**Keywords:** colocalization, condensates, Notch, transcription

## Abstract

Biomolecular condensates are a quickly emerging area of research that strongly impacts how we view the inner workings of the cell itself. Here, we explore the connection between the Notch signaling pathway and nuclear condensate localization. In this study, we hypothesize that activated Notch intracellular domain (NICD) fragments differentially localize into unique nuclear condensates. Using both overexpression and endogenous systems, our results reveal that N1ICD, N2ICD, and N4ICD efficiently localize into subnuclear bodies, whereas N3ICD inefficiently localizes into similar subnuclear puncta. These “notchosomes” appear to be unique because they do not colocalize with markers for other known nuclear puncta. In contrast, we find that N1ICD does colocalize with other known Notch‐interacting proteins, including MAML‐1, RBPj, AES1, and SKIP1, but not NACK1. Through deletion analysis of the N1ICD C‐terminal tail, we identify multiple regions of mouse N1ICD that are necessary for localization into notchosomes, including sequences immediately C‐terminal to the ankyrin domain and sequences within the transactivation domain. We also show that N1ICD localization into notchosomes may be important for N1ICD transcriptional activity from some, but not all, Notch‐responsive promoters. Collectively, our results show that Notch NICD domains form nuclear localized biomolecular condensates that may be important for transcriptional activity.

## Graphical abstract

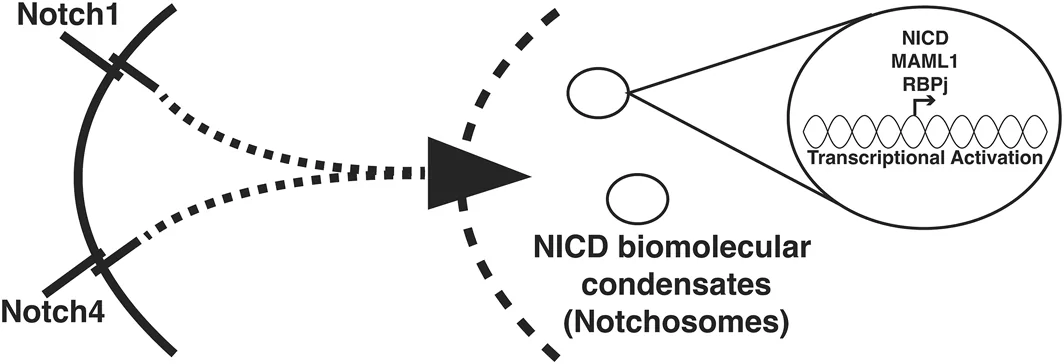

## 1 INTRODUCTION

The Notch signaling pathway is evolutionarily conserved in all multicellular animals,^1^ functioning to control cell differentiation during development, angiogenesis, and T‐cell maturation.^2^ The dysregulation of this pathway leads to many health issues, such as T‐cell acute lymphoblastic leukemia (T‐ALL) and acute lymphoblastic leukemia (ALL).^2^ Understanding the mechanisms of Notch and how it is regulated could lead to future clinical interventions.

Notch receptors are activated when Notch ligands (Jagged‐1 and ‐2, and Delta‐like‐1, ‐3, and ‐4),^3^, ^4^, ^5^, ^6^, ^7^ on a neighboring cell directly interact with Notch receptors. Endocytosis of Notch ligands generates a pulling force that causes a conformational change in the receptor, opening it for cleavage at the S2 site, followed by another cleavage by gamma‐secretase at the S3 site inside the cell membrane.^8^ This action releases the intracellular portion of the Notch receptor, called the NICD (Notch intracellular domain). The NICD travels into the nucleus, where it forms a tripartite complex with MAML and RBPJ to activate downstream target genes, such as the Hes and Hey family of genes^9^, ^10^ which are important regulators of cell differentiation and development.^11^, ^12^ There are four homologs of the Notch protein in mammals (Notch1–4) that are part of this canonical pathway, with Notch1 and Notch2 found to be more similar in structure than Notch3 and Notch4.^13^ When translocating to the nucleus, the Notch1 NICD (N1ICD) forms nuclear puncta‐like structures appearing to be non‐membrane‐bound organelles, otherwise known as condensates.^14^, ^15^

There are many well‐categorized condensates throughout the cell, both in the cytoplasm and the nucleus, including nuclear speckles, P‐bodies, stress granules, nucleoli, and Cajal bodies, among others.^16^ Lacking a formal barrier, condensates form through liquid‐liquid phase separation (LLPS),^16^ a phenomenon where molecule–molecule interactions are more energetically favored over molecule–water interactions due to the molecules' decreased solubility in water. This induces the formation of densely packed protein‐ and nucleic acid‐containing compartments to equalize the chemical potential between both phases. Condensates allow cells to maintain concentration gradients within these phases without extra expenditure of energy; however, certain molecules are still able to diffuse across this thermodynamic barrier. This decrease in molecular solubility can be due to multivalency (the ability of one molecule to bind several molecules), either through modular protein interactions^17^ or intrinsically disordered regions (IDRs) that have multiple properties, such as DNA/RNA‐binding domains and groupings of positively or negatively charged amino acids^18^ that can lead to condensate formation. The function of these unique structures has yet to be fully explored; however, theories can be made based on their characteristics. Possible functions of condensates include increasing molecular reaction kinetics through high‐concentration areas, decreasing reactions through molecular crowding, protein sequestration, increasing the likelihood of specific molecular interactions, and regulating the molecular environment inside the cell.^16^

Although recent studies have introduced the idea that the NICD domain of Notch1 can form nuclear condensates, the purpose of this study was to solidify the evidence of localization of all the Notch isoforms into nuclear puncta or “notchosomes” and to explore the characteristics of these structures. Using both transient transfection and endogenous expression systems, we have determined that all four mammalian NICDs colocalize into notchosomes; however, the function of notchosomes remains unclear.

## 2 METHODS

### 2.1 Cell culture

HEK293T cells were cultured in Dulbecco's modified Eagle's medium (Corning) supplemented with 10% EqualFETAL (Atlas Biologicals) and 1x penicillin‐streptomycin solution. HMEC‐1 cells were cultured in endothelial cell basal medium (PromoCell) supplemented with the PromoCell Supplement Pack (C‐37320 fetal calf serum 0.02 mL/mL, C‐30120 endothelial cell growth supplement 0.004 mL/mL, heparin 90 μg/mL, C‐30220 recombinant human epidermal growth factor 0.1 ng/mL, C‐30310 recombinant human basic fibroblast growth factor 1 ng/mL, and C‐31061 hydrocortisone 1 μg/mL). Cells were cultured on 10‐cm plates and passaged at 80%–90% confluence. Pluripotent mouse mesenchymal stem cells (MSC), capable of differentiating along osteogenic and adipogenic pathways, were cultured in IMDM, supplemented with 10% fetal bovine serum (FBS) and 1% penicillin/streptomycin (P/S). All cells were maintained at 37°C and 5% CO_2_.

### 2.2 Plasmids and mutagenesis

The 3xFlag‐NICD constructs were gifts from Raphael Kopan^19^ (Addgene plasmids N1ICD #20183, N2ICD #20184, N3ICD #20185, N4ICD #20186). All constructs have an N‐terminal 3xFlag tag and produce the intracellular domain of the mouse Notch proteins. The NICD coding regions were then subcloned into pcDNA3.1/Myc‐His (Invitrogen) to add a C‐terminal Myc‐His tag.^20^ For the transcriptional reporter assays, Hes5‐Luc (Addgene #41724) was gifted from Ryoichiro Kageyama and Raphael Kopan.^21^ This luciferase reporter contains portions of the native mouse Hes5 promoter and includes two RBPJ‐binding sites to report Notch NICD transcriptional activity. The Hes4‐Luc reporter (−139 to −9) was cloned by PCR amplification of the ChIP‐isolated Hes4 DNA. This PCR fragment was cloned into pGL3‐Basic (Promega) with 5′ Kpn1 and 3′ SacI sites.^22^ The 4xCSL‐Luc promoter was a gift from Raphael Kopan^23^ (Addgene #41726). The CMV‐β‐gal plasmid was originally from Clontech.

The serial truncations of N1ICD were made using the 3xFlag‐N1ICD plasmid and Gibson Assembly. PCR was used to amplify the entire plasmid, using oligos designed to exclude the codons for the 70‐amino acid sections removed for each truncation as specified in Table S1.

The Flag‐N1ICD_Δ2111‐2181_ plasmid was made by deletion and ligation of the 70 amino acids directly C‐terminal of the ankyrin domain in the 3xFlag‐N1ICD construct described above. The N3ICD_+N1 2111‐2181_ plasmid was created using Gibson Assembly, cloning amino acids 2111–2181 from N1ICD and inserting them in frame directly C‐terminal of the ankyrin domain in 3xFlag‐N3ICD, leaving the original 3xFlag‐N3ICD C‐terminal domain attached.

The constructs used in the colocalization studies with the proteins of the tripartite complex were gifts from Yanan Yang (pCMV10‐RBPJ Addgene plasmid #84601)^24^ and Brandon J. White (MAML‐Myc)^25^ using the protocol of Fryer et al.^26^ The condensate marker plasmids were gifts from Gregory Matera (pEGFP‐coilin Addgene plasmid #36906),^27^ Wesley Sundquist and Kattharine Ullman (pLVX‐SRRM2‐mCherry Addgene plasmid #174089),^28^ and Agnel Sfeir (pcDNA3.1(Hygro) TagBFP‐hNucleolin Addgene plasmid #182592).^29^

### 2.3 Transfection, shear stress, and cisplatin treatments

Transient transfections of HEK293T cells in 24‐well plates were performed using 100 ng of various constructs and a 5:1 PEI (polyethylenimine) to DNA plasmid ratio.

For EDTA activation of endogenous Notch, HMEC‐1 cells were plated on coverslips for 48 h before the addition of 0.5‐mM EDTA for 30–60 min, with 1 h of recovery in complete endothelial cell basal medium in the incubator at 37°C and 5% CO_2_, based on the protocol from Rand et al.^30^ After recovery, cells were fixed according to the immunofluorescence protocol and washed with antibodies before imaging.

For shear activation of endogenous Notch in HMEC‐1 cells, shear stress was imitated using an orbital shaker (VWR International; VWR Standard Orbital Shaker, Model 1000, 89032‐088) placed in the incubator and shaken at a speed of about 180 rpm following the protocol from Dardik et al.^31^ Orbital shear stress was determined by using the equation $\alpha \sqrt{n\rho {\left(2\pi f\right)}^{3}}$, where *α* is the radius of rotation of the orbital shaker (1.5‐cm orbit), n is the viscosity of the medium (0.0101), ρ is the density of the medium (0.9973 g/mL), and f is the rotations/sec of the shaker (∼180 rpm). From this equation, we determined the shear stress force acting on the cells from orbital shaking to be ∼12 dyne/cm^2^. Cells were plated in 6‐cm wells with coverslips attached to the bottom of the well and incubated for 48 h to allow for any previously activated Notch by trypsin/EDTA to be cleared from cells. When used, leptomycin B was applied 3 h before shear stress stimulation at a concentration of 10 μM. Plates undergoing shear stress were then placed onto the rotator in the incubator at 37°C and 5% CO_2_ for 45 min before fixation for immunofluorescence observation or collection for Western blot analysis.

For the cisplatin treatment, we introduced cisplatin (10 μM) into the growth media of murine MSCs for 3 h,^32^ followed by a PBS wash. We immediately collected samples for the 0‐h timepoint or added fresh media and incubated overnight before collecting the 24‐h timepoint.

### 2.4 Luciferase assays

HEK293T cells were plated at 50,000 cells/well in a 24‐well plate. The next day, cells were transfected with the appropriate plasmids using a 5:1 PEI to DNA ratio in serum‐free DMEM. Cells were transfected with 100 ng/well of luciferase promoter, 10 ng/well of CMV‐β‐galactosidase for data normalization, and 50 ng/well of experimental plasmids. 48 h after transfection, cells were collected and lysed in 1x firefly luciferase lysis buffer (Biotium) before being analyzed following the manufacturer's protocol (firefly luciferase assay kit, Biotium) using a Promega© GloMax Multi Detection System luminometer. Luciferase activity was normalized to β‐galactosidase activity (Promega), and values were reported as a fold change to control. To minimize transfection variations and background, all conditions were performed in triplicate for each independent experiment.

### 2.5 Immunofluorescence and microscopy

Cells were fixed with ice‐cold methanol for 10 min at 25°C followed by three immediate washes with 1x PBS. Cells were then permeabilized with 0.1% Triton X‐100 for 10 min and blocked with 5% goat serum for 30 min at room temperature. The cells were incubated in primary antibodies overnight, followed by five washes of PBST (1x PBS, 0.1% Tween‐20), then incubated with fluorescent‐conjugated secondary antibodies (Table S2) for 30 min followed by five more washes of PBST before mounting onto glass slides with an antifade DAPI mounting media (0.5 mg/mL DAPI, glycerol, PBS, 0.02% n‐propyl‐gallate, 0.1% sodium azide). Cells were then imaged using an EVOS FL Auto microscope. Controls included nontransfected cells incubated in both primary and secondary antibodies as well as transfected cells with a no‐primary control. MSCs were imaged using a Zeiss LSM 900 confocal microscope.

### 2.6 mRNA expression analysis

After the orbital shear stress application to HMEC‐1 cells, total mRNA was collected by lysing the cells with RiboZol (VWR; RiboZol RNA Extraction Reagent, N580‐200ML). mRNA was reverse‐transcribed to complementary DNA using the High‐Capacity cDNA Reverse Transcription Kit (Thermo Fisher Scientific; High‐Capacity cDNA Reverse Transcription Kit, 4368814). qRT‐PCR analysis was then applied using a qPCR master mix (Biotium; Fast EvaGreen qPCR Master Mix, 31003‐1) with oligos specified for human mRNA. The dependent variable genes were folded to the housekeeping gene β‐actin, and the independent variable, shear stress, was folded to the static control.

### 2.7 Western blots

Prepared protein lysate samples were separated through SDS‐PAGE gels. Samples were transferred onto nitrocellulose membranes and blocked in TBS‐T (140‐mM NaCl, 25‐mM Tris‐HCl, pH 7.4, 0.1% Tween‐20) with 5% bovine serum albumin. Membranes were incubated with primary antibody (1:1000 αNotch1 C‐10 (Santa Cruz), 1:500 αCleaved Notch1 D3B8 (Cell Signaling), 1:1000 αVinculin (Santa Cruz)) overnight on a rotator at 4°C. After incubation, membranes were washed 3 × 10 min in TBS‐T and then incubated with a secondary antibody solution composed of horseradish peroxidase‐conjugated secondary antibodies at a concentration of 1:10,000. Membranes were then washed again 3 × 10 min in TBS‐T with a quick wash in 1x TBS, and proteins were detected through chemiluminescence.

### 2.8 Identification of potential IDRs in Notch N1ICD

Both flDPnn^33^ and AlphaFold3^34^ were used to analyze the IDRs of mouse N1ICD (residues 1744–2531). The AlphaFold3‐derived structure was aligned with the known structure of the ankyrin repeats (PDB ID: 2QC9) of mouse N1ICD using MatchMaker in ChimeraX,^35^ providing validation of the predicted structure. The root‐mean‐square deviations (RMSDs) for the alignments of chains A and B of the ankyrin repeat structure were 0.719 Å and 0.991 Å, respectively. ChimeraX was also used to visualize the AlphaFold3 structure with coloring based on the predicted local distance difference test (pLDDT) scores. Additionally, five AlphaFold3‐predicted structures were aligned using MatchMaker in ChimeraX to highlight regions of well‐defined structure and those with intrinsic disorder (Figure S1).

## 3 RESULTS

### 3.1 Literature review of Notch‐containing nuclear puncta

In 2007, Hooper et al., observed that Notch1 NICD domains (N1ICD) localized into subnuclear bodies in SH‐SY5Y neuroblastomas, HEK293 cells, CHO cells, and primary rat cortical neurons,^14^ raising the hypothesis that Notch NICD domains might form non‐membrane‐bound nuclear organelles. Based on this hypothesis and the emerging understanding of non‐membrane‐bound organelles, we performed a literature search to determine whether NICD‐containing nuclear puncta had been observed in other publications but not specifically reported as puncta. We focused on manuscripts that used immunofluorescence to monitor NICD cellular localization to gain a nonbiased understanding of NICD staining patterns in a variety of cell lines and conditions. As summarized in Table 1, we observed NICD molecules forming nuclear puncta in many but not all reviewed manuscripts. Moreover, we found evidence that in addition to Notch1, other Notch isoforms can also form nuclear puncta. These observations imply that NICD molecules can form nuclear puncta not only in transfected cell lines, but also under endogenous expression in various cell lines and even in some tissues. Based on these findings, we set out to directly test the hypothesis that NICD molecules form nuclear puncta and whether these puncta might be biomolecular condensates.

**TABLE 1:** Literature review of Notch immunofluorescence and nuclear puncta.

Authors	Title	Cell line	Tag	Antibody	Puncta?	Fig #
Ribeiro et al.^36^	Notch1 and galectin‐3 modulate cortical reactive astrocyte response after brain injury	Primary astrocytes	None; endogenous	Anti‐Notch1	Yes	1
Chiappara et al.^37^	Cigarette smoke upregulates the Notch‐1 signaling pathway and promotes lung adenocarcinoma progression	A549	None; endogenous	Anti‐Notch1	Yes	3
Nakano et al.^38^	Mucosal mast cell–specific gene expression is promoted by interdependent action of Notch and TGF‐β signaling	BMMC	Flag‐Notch2	Anti‐Flag	Yes	7
Saini et al.^39^	Nucleolar localization of the Notch4 intracellular domain underpins its regulation of the cellular response to genotoxic stressors	MDA‐MB‐231 AND 293T	Endogenous N4ICD	Anti‐Notch4	Yes	1,3,4
Kretschmer et al.^40^	Matrix stiffness regulates Notch signaling activity in endothelial cells	HUVEC	None; endogenous	Anti‐cleaved Notch1 Val1744	No	1
Fernandez‐Martinez et al.^41^	Attenuation of Notch signaling by the Down syndrome‐associated kinase DYRK1A	293T and C2C12 Endogenous	Endogenous Notch1; Myc	Anti‐cleaved Notch1; anti‐Myc	Yes	6
MacKenzie et al.^42^	Notch4 inhibits endothelial apoptosis via RBP‐Jk‐dependent and ‐independent pathways	HMEC	HA	Anti‐HA	No	3
Beatus et al.^43^	The origin of the ankyrin repeat region in Notch intracellular domains is critical for regulation of HES promoter activity	C2C12	HA; Myc	Anti‐HA; anti‐Myc	No	5
Kuo et al.^44^	JARID1B expression plays a critical role in chemoresistance and the stem cell‐like phenotype of neuroblastoma cells	SK‐N‐BE(2)	None; endogenous	Anti‐Notch1	Yes	6
Tan et al.^45^	Notch1 signaling antagonizes the transforming growth factor‐β pathway and induces apoptosis in rabbit trophoblast stem cells	Rabbit TSCs	Endogenous Notch1; flag	Anti‐Notch1; anti‐Flag	Yes	2
Breunig et al.^46^	Notch regulates cell fate and dendrite morphology of newborn neurons in the postnatal dentate gyrus	Dentate gyrus tissue	None; endogenous	Anti‐Notch1	Yes	1
Marcel et al.^47^	Notch1 regulates autophagy controls survival and suppressor activity of activated murine T‐regulatory cells	T‐regulatory cells	None; endogenous	Anti‐Notch1; anti‐cleaved Notch1	Yes	4
Mori et al.^48^	Notch3‐Jagged signaling controls the pool of undifferentiated airway progenitors	Undifferentiated airway progenitors	Endogenous Notch1, Notch2, and Notch3; HA	Anti‐cleaved Notch1; anti‐Notch2; anti‐Notch3; anti‐HA	Yes, N3ICD only	2
Foran et al.^49^	Notch1 phase separation coupled with percolation facilitates target gene expression and enhancer looping	SH‐SY5Y, HEK293, HeLa, and U2OS cells	Endogenous Notch1	Anti‐Notch1	Yes	1

### 3.2 NICD molecules form nuclear condensates

Based on our literature review, we sought to confirm the presence of Notch nuclear puncta. We first transfected an N1ICD‐eGFP fusion protein construct into HEK293T and HMEC‐1 endothelial cells to observe Notch nuclear puncta in live cells and found that N1ICD‐eGFP localized into nuclear puncta in both HMEC‐1 and 293T cells (Figure 1A,B). To determine whether these subnuclear bodies are condensates formed through LLPS, we treated transfected 293T cells with 4% 1,6‐hexanediol. The aliphatic alcohol 1,6‐hexanediol is capable of dissolving LLPS condensates through interruption of the weak hydrophobic interactions necessary for condensate function but does not disrupt structures or condensates reliant on electrostatic interactions. As shown in Figure 1A, 1,6‐hexanediol successfully dissolved the N1ICD‐eGFP nuclear puncta in living cells, suggesting these puncta are formed by LLPS. To determine whether these puncta have the fluid‐like property of biomolecular condensates, we performed live‐cell imaging of N1ICD‐eGFP‐transfected 293T cells. Consistent with the idea that these puncta are biological condensates, N1ICD puncta were observed to undergo fusion and separation (Figure 1C). Similarly, Foran et al.^49^ used FRAP analysis to demonstrate that N1ICD‐containing nuclear puncta are fluid and found that purified N1ICD proteins spontaneously form condensates in vitro. Taken together, these results support the hypothesis that N1ICD forms nuclear condensates through an LLPS mechanism.

**FIGURE 1:**
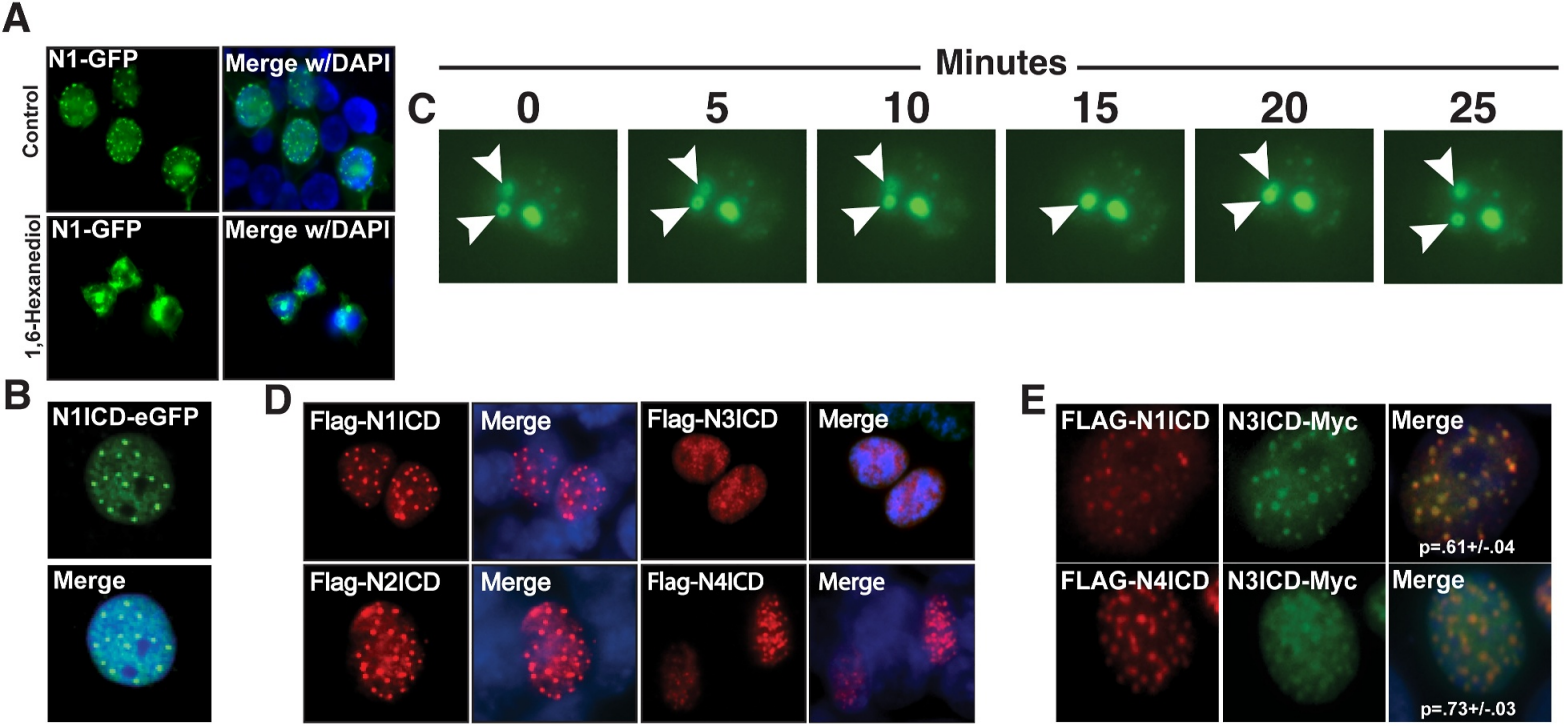
Notch isoforms differentially form nuclear condensates. (A) Live 293T cells were transiently transfected with N1‐GFP and treated with 4% 1,6‐hexanediol for 15 min to disperse nuclear condensates. (B) Live HMEC endothelial cells were transiently transfected with cDNA encoding N1ICD‐eGFP fusion protein. (C) Live‐cell imaging of 293T cells transfected with N1ICD‐eGFP and imaged every 5 minutes. (D) 293T cells were transfected with either Flag‐N1ICD, Flag‐N2ICD, Flag‐N3ICD, or Flag‐N4ICD and detected with an anti‐Flag antibody before fluorescent imaging. (E) 293T cells were transfected with either Flag‐N1ICD and N3ICD‐Myc or Flag‐N4ICD and N3ICD‐Myc and imaged by immunofluorescence with anti‐Flag and anti‐Myc antibodies. All panels show representative images from experiments that were performed at least three independent times.

In addition to Notch1, our literature review also identified instances where other NICDs also form nuclear puncta. Based on this, we next asked whether any of the other Notch isoforms can form condensates when transfected into 293T cells. We individually transfected cDNAs encoding Flag‐tagged versions of each NICD into 293T cells and used immunofluorescent microscopy to visualize NICD‐containing puncta. As shown in Figure 1D, we found that not only did N1ICD localize into nuclear condensates, but so did N2ICD and N4ICD. N3ICD puncta were markedly different compared to the other puncta. N3ICD appeared to form many small puncta that combined to form a nearly uniform background. Interestingly, when N3ICD was co‐transfected with either N1ICD or N4ICD, the NICDs appeared to colocalize in puncta, suggesting that N3ICD may rely on other NICDs to form puncta (Figure 1E). These results not only demonstrated that multiple NICD isoforms can form puncta but also suggested that different NICD isoforms may have varying capacity to form nuclear condensates. Moreover, the apparent ability of N3ICD to form puncta when co‐transfected with N1ICD or N4ICD suggests that interactions between NICD molecules might help some NICDs to enter puncta, which is consistent with our previous observations.^20^

### 3.3 N1ICD and N4ICD form nuclear puncta in endogenous conditions

To confirm that the NICD‐containing nuclear puncta were not an artifact of overexpression or GFP tags, we monitored NICD puncta formation in endothelial cells before or after exposure to shear stress, which has been shown to activate endogenous Notch signaling.^50^ To first confirm that Notch signaling is activated by shear stress in HMEC endothelial cells, confluent cultures of HMEC cells were rotated on an orbital shaker to expose them to shear forces of approximately 12 dynes for 0–90 min, and Western blotting was used to monitor N1ICD accumulation in whole‐cell lysates. As shown in Figure 2A, immunoblotting with antibodies that specifically detect N1ICD cleaved at the S3 position (Val1744) revealed a steady increase of N1ICD from 0 to 45 min, followed by a gradual decrease from 60 to 90 min. In the early timepoints, N1ICD blotted as two bands, which largely resolved to primarily a single higher‐molecular weight species in the later timepoints. Immunoblotting with antibodies that detect the intact Notch1 receptor showed no change in Notch1 receptor expression, confirming Notch activation by S3 cleavage rather than increased Notch1 expression. To confirm that shear stress also activates Notch transcriptional activity in HMEC cells, we used RT‐PCR to monitor mRNA expression of Notch‐responsive genes (Hey1, Hes1, Hey2) and Krüppel‐like factor 2 (Klf2), a known shear stress‐responsive gene,^51^ after exposure to 90 min of shear stress. As shown in Figure 2B, shear stress‐initiated transcription of the positive control Klf2 mRNA, as well as the Hey1, Hes1, and Hey2 mRNAs, confirmed that Notch activated by shear stress is transcriptionally active. Having established that shear stress activates both N1ICD cleavage and transcriptional activity, we next asked if shear stress also induced NICD puncta formation. To accomplish this, coverslips were attached to the outside edge of cell culture dishes over which HMEC‐1 cells were cultured and allowed to grow to confluence before exposure to 45 min of orbital shear stress. After shear stress, coverslips were removed and processed for immunofluorescent microscopy. As shown in Figure 2C, N1ICD‐containing puncta accumulated in both the nuclear and perinuclear compartments. In comparison, N4ICD accumulated in bright nuclear puncta with little or no perinuclear staining. To demonstrate endogenous puncta formation in a second cell line, we treated MSC with cisplatin, a DNA‐damaging chemotherapeutic agent, which has been shown to upregulate Notch1 signaling.^52^ As shown in Figure 2D, MSCs treated with cisplatin strongly induced the formation of N1ICD‐containing nuclear puncta, thus demonstrating that N1ICD puncta form in a variety of cell lines under different stress conditions.

**FIGURE 2:**
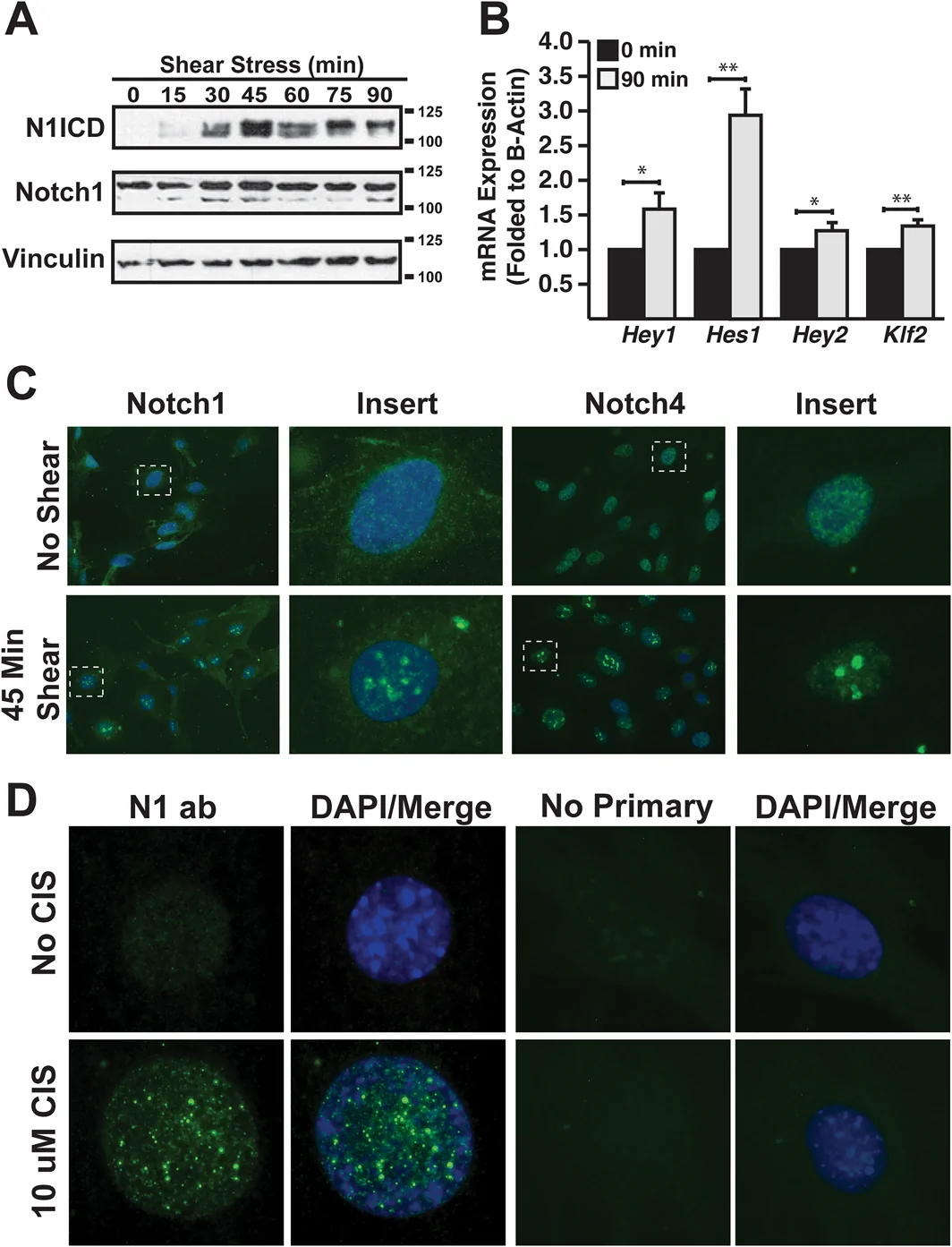
Endogenous N1ICD and N4ICD form condensates. (A) HMEC‐1 cells were exposed to ∼12 dynes/cm^2^ of orbital shear stress, and whole‐cell lysates were Western blotted with anti‐Val1744 antibodies to specifically detect N1ICD or with antibodies against the N1ICD C‐terminal to detect cleaved and uncleaved N1ICD. Western blotting with anti‐vinculin antibodies was used to monitor protein loading. (B) HMEC cells were exposed to ∼12 dynes of shear stress for 90 min, and RT‐PCR was used to monitor the expression of Notch‐responsive genes (Hey1, Hes1, and Hey2) or the known shear‐induced Krüppel‐like factor 2 mRNA. Values are mean +/− SEM from five independent experiments. * = *p* < 0.05, ** = *p* < 0.01. (C) HMEC‐1 cells were subjected to 0–45 min of shear stress before fixation and imaging with antibodies against the N1ICD and N4ICD C‐terminal domains. Shown are representative images from a single experiment that was performed three independent times. (D) Mesenchymal stem cells were treated with 10‐μM cisplatin, and anti‐N1ICD C‐terminal antibodies were used to monitor N1ICD by immunofluorescence. No primary (secondary only) controls were used to monitor the specificity of antibody staining. Shown are representative images from a single experiment that was performed three independent times with similar results.

Collectively, these results agreed with the results of Figure 1 and demonstrated that N1ICD and N4ICD form nuclear biomolecular condensates after endogenous activation of Notch signaling. Based on these findings, we propose the name “notchosome” to describe NICD‐containing nuclear puncta.

### 3.4 Notchosomes don't localize with known nuclear body markers

To gain insight into the nature of notchosomes, we first asked if N1ICD colocalized with markers for other known nuclear condensates. We co‐transfected 293T cells with cDNAs encoding N1ICD and either the Cajal body marker coilin,^53^ the nuclear speckle marker SRRM2,^54^ or the nucleolar marker nucleolin.^55^ As shown in Figure 3, N1ICD did not colocalize with any of these markers, suggesting notchosomes may be unique nuclear organelles. In support of this, Hooper et al. previously showed that N1ICD does not colocalize into PML bodies.^14^ Previous work by Sonoshita et al.^15^ demonstrated that N1ICD can colocalize with the amino‐terminal enhancer of split (AES) protein in HCT116 colorectal cancer cell nuclear puncta. To determine if AES also colocalized with N1ICD in 293T cells, we transfected GFP‐tagged AES with or without N1ICD and monitored cellular localization by fluorescent microscopy. As shown in Figure 3, AES weakly formed nuclear puncta on its own, but puncta formation was enhanced by co‐transfection with N1ICD. Although not exhaustive of all nuclear condensates, these results suggest that notchosomes may be unique condensates.

**FIGURE 3:**
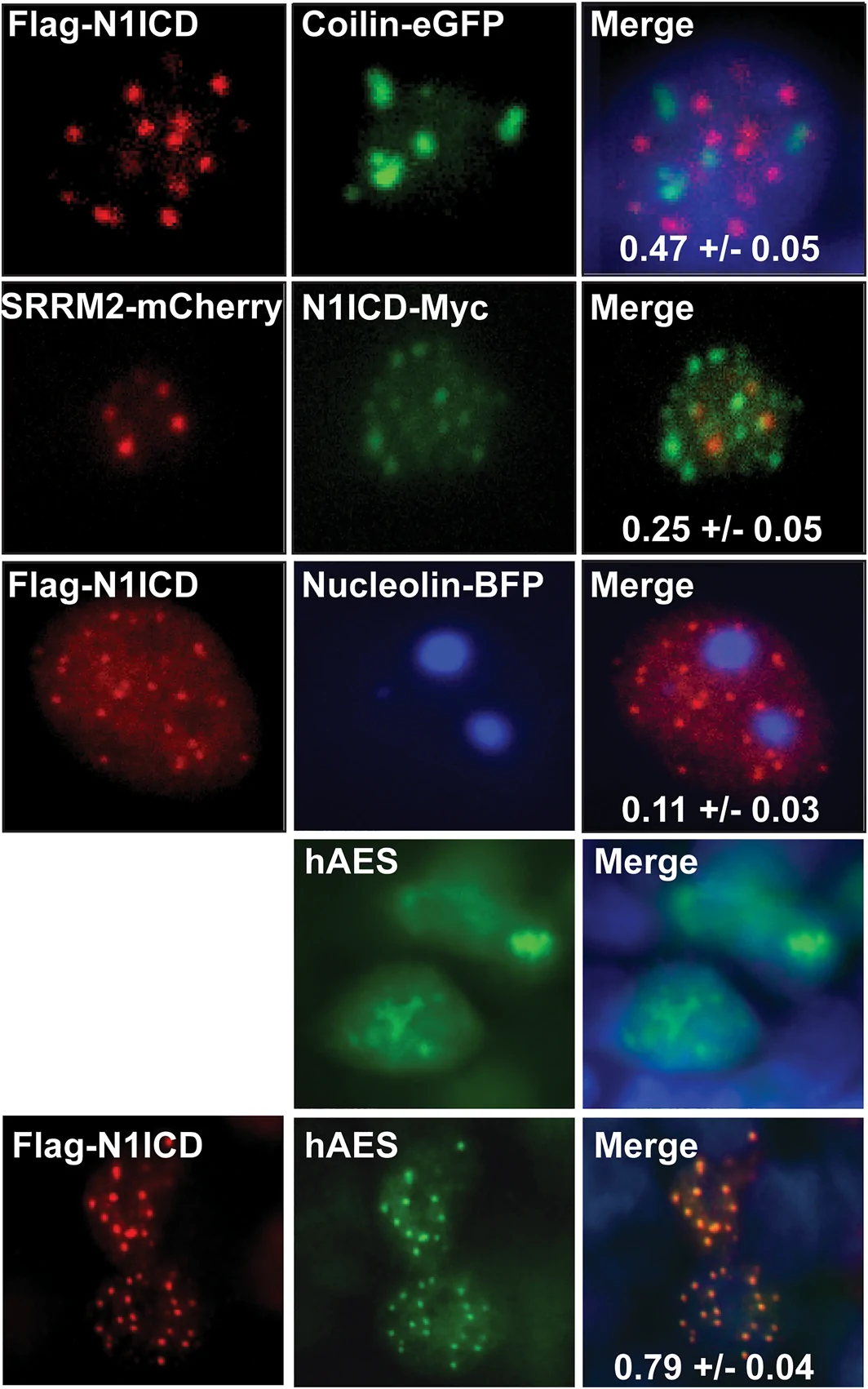
Notchosomes do not localize with known nuclear body markers. 293T cells were transfected with cDNAs encoding N1ICD and either eGFP‐tagged coilin (Cajal bodies), Myc‐tagged SRRM2 (nuclear speckles), BFP‐tagged nucleolin (nucleolus), or eGFP‐tagged AES to detect colocalization between N1ICD and Cajal bodies, nuclear speckles, or nucleoli.

### 3.5 Notchosomes contain other known Notch‐interacting proteins

To determine if notchosomes contain other proteins associated with Notch transcriptional function, we performed colocalization studies between N1ICD and other members of the canonical Notch tripartite complex RBPj and MAML1.^13^ In agreement with previous observations^56^, ^57^, ^58^ Figure 4A shows that MAML1 formed condensates on its own and colocalized into condensates with N1ICD. Colocalization between N1ICD and MAML1 was measured by Pearson correlation coefficient (PCC) analysis, which indicated strong colocalization (PCC = 0.79+/−0.05). Unlike MAML1, RBPj only loosely localized to notchosomes. Instead, the majority of RBPj evenly dispersed throughout the nucleus. Moreover, transfection of RBPj appeared to predominantly dissolve notchosomes, resulting in NICD proteins being more evenly dispersed throughout the nucleus (Figure 4B). In addition to the canonical members of the Notch tripartite complex, other noncanonical proteins, including ski‐interacting protein (SKIP) and Notch activation complex kinase (NACK), have also been shown to interact with NICD proteins,^59^, ^60^ and we asked if these proteins might also localize in notchosomes. We found that SKIP weakly formed nuclear puncta when transfected alone and colocalized to notchosomes when co‐transfected with N1ICD (PCC = 0.82+/−0.03) or N3ICD (PCC.66+/−0.04) (Figure 4C). In comparison, NACK did not localize to puncta, remained in the cytoplasm, and did not localize to notchosomes when co‐transfected with N1ICD (Figure 4D). Taken together, these results demonstrated that notchosomes contain at least two members of the canonical tripartite complex, suggesting notchosomes may play a role in Notch transcriptional activity.

**FIGURE 4:**
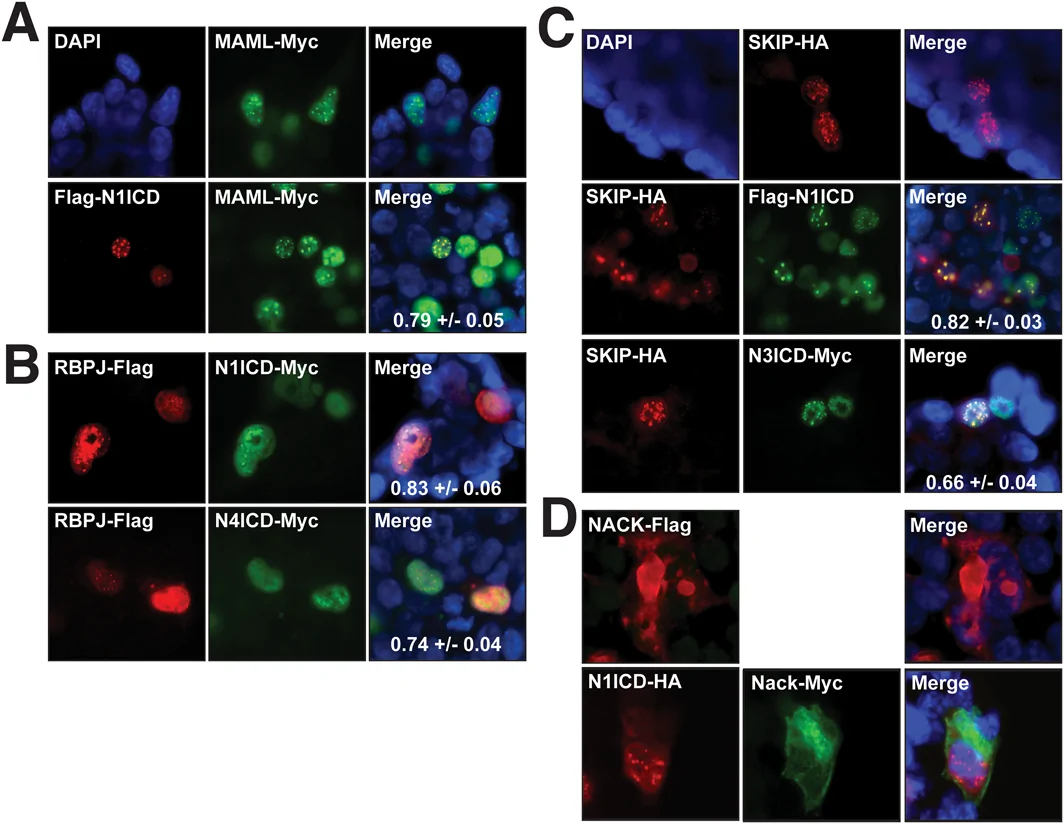
Notchosomes contain other known Notch‐interacting proteins. (A) 293T cells were transfected with cDNA encoding Myc‐tagged MAML +/− Flag‐tagged N1ICD or N3ICD. (B) 293T cells were transfected with cDNAs encoding Flag‐tagged RBPj +/− Myc‐tagged N1ICD. (C) 293T cells were transfected with cDNA encoding HA‐tagged ski‐interacting protein +/− Flag‐tagged N1ICD or N3ICD. (D) 293T cells were transfected with Flag‐tagged Notch activation complex kinase +/− HA‐tagged N1ICD. In all panels, protein localization was monitored with anti‐Flag, Myc, or HA antibodies. Shown are representative images from single experiments that were performed at least three independent times. Pearson correlation coefficient values were calculated from at least four individual cell images with ImageJ software using the JACoP plugin.

### 3.6 Notch contains C‐terminal IDRs

IDRs are one of the factors important to the formation of LLPS condensates.^16^ IDRs within N1ICD have previously been observed in the RAM domain and in sequences C‐terminal to the ankyrin domain.^49^, ^61^ To confirm these findings, we used flDPnn^33^ and AlphaFold3^34^ to analyze N1ICD for IDRs. In agreement with previous findings, flDPnn predicted a small IDR within the RAM domain and extensive IDRs in the C‐terminal region (Figure 5A). These predictions were corroborated by AlphaFold3 predictions, which show low confidence (i.e., low pLDDT scores) in the regions N‐terminal and C‐terminal of the ankyrin domain (Figure 5 and Figure S1). However, both flDPnn and AlphaFold3 found a region between 2301 and 2308 that is less disordered than the surrounding sequences, suggesting there may be a helical element between amino acids 2301 and 2308 of the mouse N1ICD. Interestingly, the disorder predictions of the mouse N1ICD are similar to IDRs predicted in the *Drosophila* NICD^62^ by metapredict^63^ although the flDPnn and AlphaFold3 predictions were more conservative. Further comparison of human, sponge, fly, and hagfish NICD domains by ClustalW and flDPnn suggest that even though the N‐terminal and C‐terminal domains flanking the ankyrin domains (as defined by Lubman et al.^64^) have scarce sequence homology, the IDRs in these domains appear to be conserved (Figure [Link], [Link]). Based on this analysis and the findings of Hooper et al.^14^ that determined the region C‐terminal of amino acid 2094 (the end of the ankyrin domain) was important for condensate formation, we proceeded to construct a series of plasmids that progressively truncated the N1ICD C‐terminal. Deletion of the most C‐terminal 210 amino acids (AA 2531–2321), which encompasses the N1ICD PEST domain and the largest IDR nearest to the C‐terminus, had little effect on notchosome formation (Figure 5B). Notchosome formation was significantly disrupted by the further deletion of 280 amino acids (2531–2251) and 350 amino acids (2531–2181). Notably, amino acids between 2391 and 2181 correspond to the N1ICD transcriptional activation domain (TAD), suggesting that the TAD may be critical for notchosome formation. Deletion of the full 420 amino acid C‐terminal domain (2531–2111) did not cause further change in condensate formation; however, because condensates were already absent in the 280‐amino acid deletion, we worried that amino acids important for condensate formation between amino acids 2111 and 2181 would not be detected and therefore created an additional deletion encompassing only the region between 2111 and 2181, which almost entirely prevented condensate formation (Figure 5B). This region contains several interesting features, including a small IDR, the serine‐threonine‐rich (STR) region,^65^ and one of the N1ICD nuclear localization signal (NLS) domains. Importantly, N1ICD still localized to the nucleus, as it has two NLS domains with the first one being located N‐terminal to the ankyrin domain.^66^ Despite the inability of the 2111–2181 deletion construct to form notchosomes on its own, when co‐transfected with another NICD (N1ICD‐eGFP), both constructs colocalized in notchosomes, suggesting again that molecular interactions between NICDs may be an important determinant of notchosome formation (Figure 5C). Overall, these results suggested that there are at least two regions in the N1ICD C‐terminal that are important for condensate formation, with the first between amino acids 2321 and 2251 in the TAD domain and the second directly C‐terminal of the N1ICD ankyrin domain between amino acids 2181 and 2111.

**FIGURE 5:**
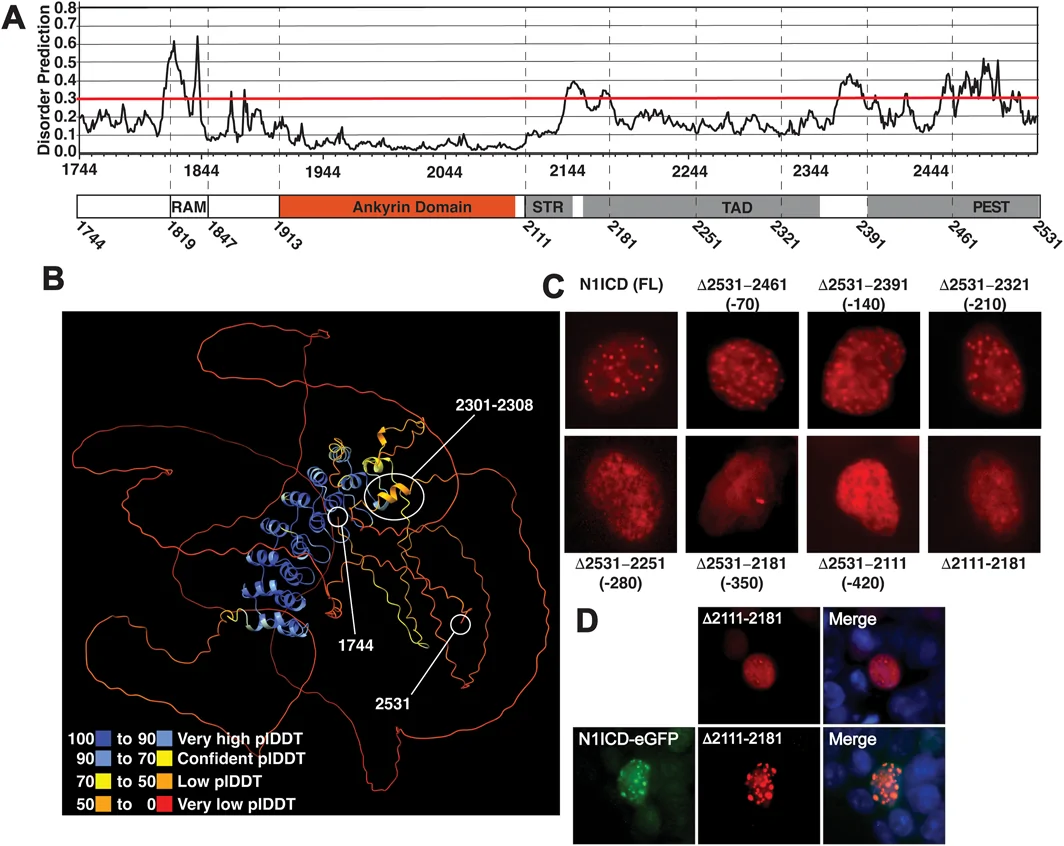
Two regions C‐terminal of the ankyrin domain are important for condensate localization. (A) Positions of N1ICD intrinsically disordered region domains predicted by flDPnn (above dotted line) and various C‐terminal deletions. (B) AlphaFold3 prediction of N1ICD showing N‐terminal (1744), C‐terminal (2531), predicted small α‐helix between 2301 and 2308, and associated confidence (pLDDT scores). (C) 293T cells were transfected with Flag‐tagged truncations of N1ICD and imaged by immunofluorescence using anti‐Flag antibodies. (D) 293T cells were transfected with mutant Flag‐N1ICD lacking amino acids 2111–2181 or co‐transfected with Flag‐N1ICD‐eGFP. All panels in C and D show representative images from experiments that were performed at least three independent times.

### 3.7 Transcriptional activity of Notchosomes

Given the well‐known transcriptional role of Notch proteins, it was important to determine whether NICD localization to notchosomes correlated with transcriptional activity. To address this, we used luciferase reporter assays in transiently transfected 293T cells to compare the transcriptional activity of NICDs when they were localized either inside or outside of notchosomes. We focused on the region between amino acids 2111 and 2181 of N1ICD because deletion of this region mostly ablated condensates while minimally impacting the transcriptional activation domain (TAD) located approximately between AAs 2172–2345.^67^ As shown in Figure 6, deletion of 2111–2181 decreased transcriptional activity of N1ICD on the Hes5 and 4xCSL promoters, although the N1ICD mutant still maintained transcriptional activity compared to N1ICD with a full C‐terminal deletion (CTD). In comparison, the N1ICD mutant only trended down on the Hes4 promoter, and surprisingly, we were not able to detect any significant effect on the Hes1 promoter. This result suggested that N1ICD may be more transcriptionally active when localized inside of notchosomes, but given that deletion of 2111–2181 reduced transcriptional activity twofold at most on the Hes5 promoter, it is unclear how significant this result is.

**FIGURE 6:**
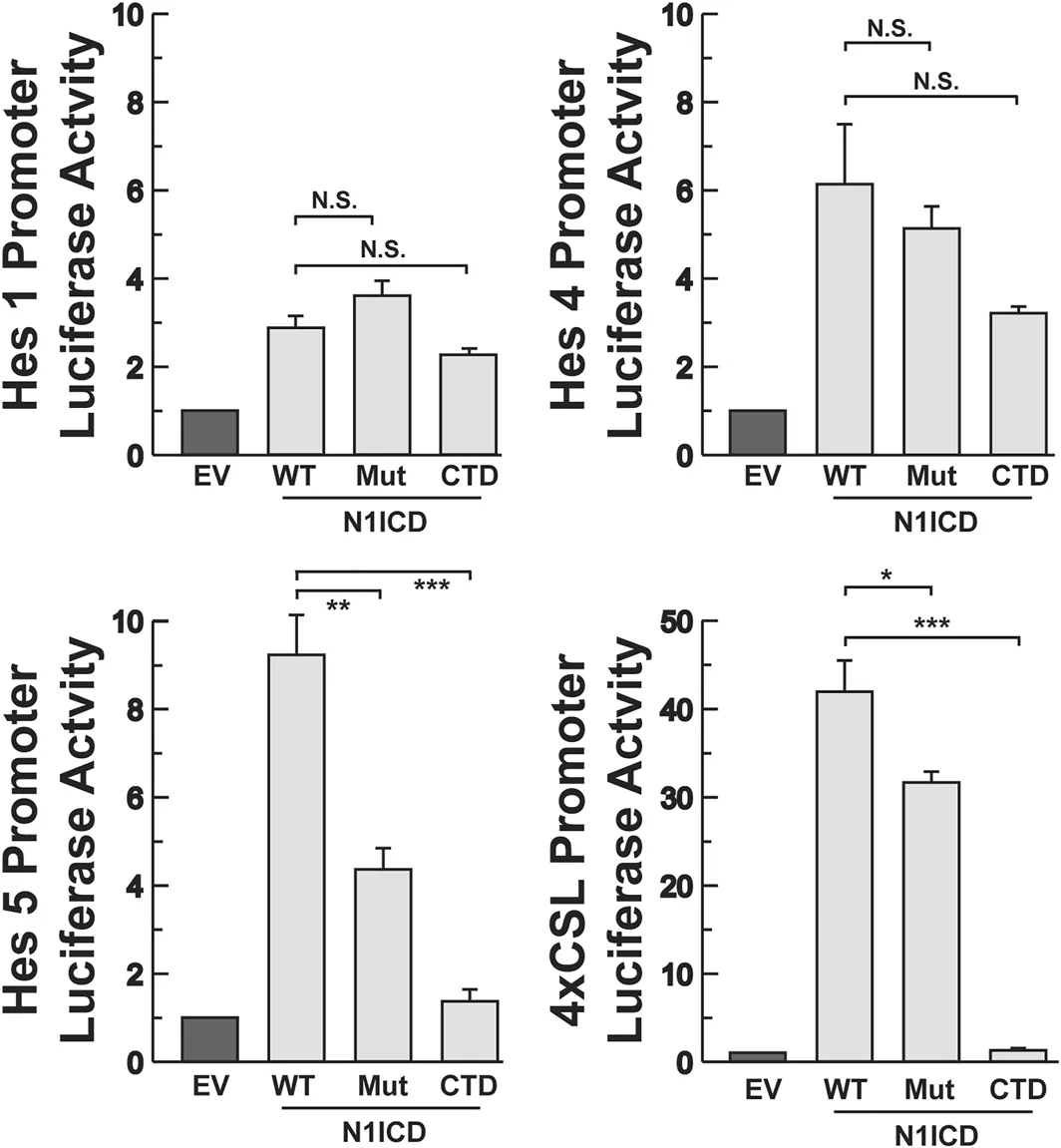
Transcriptional activity of notchosomes. 293T cells were co‐transfected with various Notch‐responsive promoters (Hes1, Hes4, Hes5, or 4xSCL) and either empty vector, wild‐type N1ICD (WT), mutant N1ICD lacking amino acids 2111–2181 (Mut), or N1ICD lacking the entire C‐terminal domain. Shown are the average +/− SE of three or more experiments. Statistical significance was determined through a Student's two‐tailed *t*‐test, assuming equal variances, where *** is *p* < 0.001, ** is *p* < 0.01, and * is *p* < 0.05.

## 4 DISCUSSION

In 2007, Hooper et al.^14^ first reported N1ICD localization into nuclear puncta. Given the vast literature on Notch signaling and the relatively new understanding of non‐membrane‐bound organelles, we hypothesized that Notch‐containing nuclear puncta may have been observed for many years in immunofluorescence experiments but dismissed as artifactual data. To test this hypothesis, we surveyed past literature looking for examples of immunofluorescence analysis of Notch and found many manuscripts that show Notch nuclear puncta in overexpression systems, in endogenous cell culture conditions, and in in vivo imaging experiments (Table 1). We also observed several instances of immunofluorescence experiments that did not display Notch nuclear puncta. Based on these observations, we believe that others before us have observed notchosomes but did not recognize them as biological condensates. We also suspect that Notch nuclear puncta are common but may only form under certain conditions because some previous experiments did not observe Notch puncta. Guided by the results of the literature survey, we sought to perform an analysis of Notch nuclear puncta and to compare the four mammalian Notch proteins for their ability to form nuclear puncta.

To establish a simple system to examine Notch nuclear puncta, we began by observing Notch condensate formation in transiently transfected 293T cells. Our results confirmed that all four mammalian Notch isoforms can form nuclear puncta in 293T cells, although puncta formation was not equivalent for all NICDs. N1ICD and N2ICD appeared to form larger and less numerous puncta compared to N4ICD, which appeared to form smaller and more numerous puncta. In comparison, N3ICD formed very small notchosomes that were so numerous that they formed a nearly uniform background with only a few puncta visible. Interestingly, N3ICD colocalized with other NICD isoforms in clearly defined puncta that were unlike those formed by N3ICD alone. Although we did not quantitate these observations, they were consistent and suggest that notchosome localization may represent yet another difference between the four mammalian Notch proteins. These observations raise two interesting questions. First, the differential puncta formed by the various NICDs are reminiscent of the differential transcriptional activities and promoter preferences of the various NICDs.^19^ Whether or not there is a correlation between notchosome physical dimensions/nuclear density and the transcriptional activity of various NICDs will be important to address in future experiments. Second, the relocation of N3ICD from indistinct puncta to distinct puncta when coexpressed with other NICDs such as N1ICD or N4ICD suggests that interactions between NICDs might be important for localization into notchosomes. This is also supported by the observation that N1ICD lacking amino acids 2111–2181 did not form puncta on its own but colocalized with full‐length N1ICD in notchosomes. Deciphering how this occurs and the biological relevance of it may be important to understand how NICDs are recruited into notchosomes.

Given the emerging understanding of non‐membrane‐bound organelles formed by LLPS, we hypothesized that Notch nuclear puncta (notchosomes) may be non‐membrane‐bound organelles that form by biomolecular condensation driven by the presence of intrinsically disordered domains (IDRs). In agreement with this, we found that 1,6‐hexanediol efficiently dissolved notchosomes and that N1ICD nuclear puncta undergo fusion and splitting behaviors. In support of this biomolecular condensate hypothesis, Foran et al.^49^ previously used FRAP analysis to confirm that Notch nuclear puncta are fluid and that purified N1ICD proteins spontaneously formed protein condensates, which is consistent with the idea that these puncta are formed by LLPS. Taken together, our results agree with Foran et al. that notchosomes are biomolecular condensates formed by LLPS. To support our overexpression data, we also monitored endogenous notchosome formation in HMEC endothelial cells that were exposed to shear stress to activate Notch signaling.^68^, ^69^ We found that N1ICD activation peaked after 45 min of shear, then declined until 90 min. The reason for this decline is uncertain but might be related to ubiquitination^25^ or phosphorylation^70^ events that control N1ICD protein half‐life, which is consistent with the accumulation of higher‐weight N1ICD species. An interesting observation was that although shear stress succeeded in forming Notch nuclear puncta, many of the N1ICD notchosomes were clustered in the cytoplasm surrounding the nucleus, thus demonstrating that N1ICD can also form condensates in the cytoplasm. Cytoplasmic localization of N1ICD has been observed previously. For example, N1ICD accumulation in perinuclear puncta of Treg cells was mobilized into nuclear puncta upon sirtuin inhibition^71^ and Notch NICD domains may also colocalize with mitochondria.^72^ This contrasted with our overexpression model in which transfected NICD formed entirely nuclear puncta, suggesting that endogenous Notch may require an additional step to form notchosomes, which is not required for transfected N1ICD, which does not require cleavage from the cell membrane. Alternatively, endogenously expressed Notch may be subject to post‐translational modification(s) that regulate nuclear localization and puncta formation. In comparison, N4ICD notchosomes showed greater nuclear localization even in unstimulated cells. What this observation means for Notch4 signaling is unknown; however, it suggests that Notch4 has higher basal activation than Notch1.

Biomolecular condensates commonly form as a result of intrinsically disordered domains within proteins.^16^ We and others^49^ found that N1ICD contains an IDR in the RAM domain and several IDRs in the C‐terminal domain. Interestingly, the RAM and C‐terminal IDR domains were largely conserved in mouse, human, sponge, fly, and hagfish, although there was very little conservation of amino acid sequences (Figure S1), suggesting that the IDRs may be conserved elements. Deletion analysis of the C‐terminal domain found that the largest IDR domain in the extreme C‐terminal was not required for notchosome formation. Instead, two regions corresponding to the TAD domain and a relatively small IDR encoded between amino acids 2111–2181, just C‐terminal to the N1ICD ankyrin domain, were necessary for notchosome formation. Amino acids between 2111 and 2181 of N1ICD encode a small part of the N1ICD transcriptional activation domain (TAD) that is important for assembly of the Notch transcriptional activation complex,^67^ the serine/threonine‐rich (STR) domain described by Ishitani et al.,^65^ and the C‐terminal N1ICD NLS. Our results suggest that the TAD domain might be important for notchosome formation because N1ICD protein lacking most of the TAD was not capable of forming puncta. This is inconsistent, however, with previous findings that N3ICD contains a weak TAD, whereas N4ICD does not contain a TAD,^19^ and yet N4ICD, but not N3ICD, efficiently formed notchosomes. Nonetheless, we found that N1ICD localization to notchosomes may be important for N1ICD transcriptional activity from some promoters such as Hes5 and 4XCSL, but not for others such as Hes1 and Hes4. This result is partially consistent with the results reported by Foran et al.,^49^ who have shown that NICD‐containing nuclear puncta are transcriptionally active and that Hes1, Hes5, and Hey1 mRNAs are transcribed inside of notchosomes. Although our Hes5 data is consistent with Foran et al., the Hes1 inconsistency might be explained by the use of different Hes1 promoters. Foran used a human Hes1 promoter encompassing −747 to +66, originally described by Lu et al.,^73^ whereas we used the commercially available (Addgene) mouse Hes1 promoter that encompasses −467 to +46, originally described by Nishimura et al.^21^ This will need to be addressed in future experiments but raises an important point about the interpretation of luciferase assays with transiently transfected plasmids as to whether or not transfected plasmids actually localize into notchosomes. Therefore, it will be critical in future experiments to monitor whether or not transfected luciferase reporter plasmids localize within notchosomes in order to fully understand the data generated by transcriptional reporter assays. Interestingly, a recent report by Bremer et al.^74^ shows that localization of the yeast Gcn4 transcription factor into LLPS condensates does not accurately predict transcriptional activity, suggesting that NICDs may be transcriptionally active both inside and outside of notchosomes, perhaps in a promoter‐dependent manner. Finally, our findings that MAML1 and RBPj also colocalize into notchosomes is consistent with the findings of Foran et al., and further support the transcriptional role of notchosomes.

In conclusion, the results presented in this paper expand upon our knowledge of how the Notch signaling pathway works and is regulated to accomplish its many functions. The important questions inspired from this data include determining the main function of notchosomes, whether they all perform the same function, and whether colocalization of different NICDs in notchosomes alters notchosome function. Answering these questions will help us to better understand the mechanisms of the Notch signaling pathway.

## AUTHOR CONTRIBUTIONS

A. Albig, T. Gazdik, and M. Detweiler conceived and designed the experiments. A. Albig, T. Gazdik, M. Detweiler, A. Zavala, D. Oke, and S. Sarkar performed the experiments. H. Charlier performed AlphaFold3 analysis. T. Gazdik, T. Lawton, and Jacob J. Crow constructed mutant DNA constructs. A. Albig and T. Gazdik prepared the final manuscript.

## CONFLICT OF INTEREST STATEMENT

The authors declare no conflicts of interest.

## ETHICS STATEMENT

This work was approved by the Boise State University Institutional Biosafety Committee.

## Supporting information

Supporting Information S1

Figure S1

Figure S2a

Figure S2b

Table S1

Table S2

## Data Availability

All data generated or analyzed during this study are included in this published article [and its supplementary information files]. Materials are available upon request from Dr. Albig (corresponding author).
