# Supplementary material for: Notch NICD domains form biomolecular condensates
Source: J Cell Commun Signal. 2025 Jul 26;19(3):e70039. doi: 10.1002/ccs3.70039 (PMC13674738; doi:10.1002/ccs3.70039)
Supplement: Supplementary file 1 [file CCS3-19-e70039-s001.docx]

| **Primary Antibody** | **Concentration** | **Company** | **Product Number** |
| --- | --- | --- | --- |
| Notch 1 (C-10) | 1:100 | Santa Cruz Biotechnology | Sc-373891 |
| Notch 4 (A-12) | 1:100 | Santa Cruz Biotechnology | Sc-393893 |
| Flag (DYKDDDDK Tag) | 1:300 | Cell Signaling Technology | D6W5B |
| c-Myc (9e10) | 1:100 | Santa Cruz Biotechnology | Sc-40 |
| HA tag | 1:500 | GeneTex | GTX115044 |
| Val1744 | 1:100 | Cell Signaling Technology | D3B8 |
| Vinculin | 1:500 | Santa Cruz Biotechnology | Sc-73614 |
| **Secondary Antibody** | **Concentration** | **Company** | **Product Number** |
| Alexa Fluor^TM^ 488 goat anti-mouse | 1 μg/mL | Invitrogen | R37120 |
| Alexa Fluor^TM^ 594 goat anti-rabbit | 1 μg/mL | Invitrogen | R37117 |

Supplemental Tabel 2:
