# Supplementary material for: Notch NICD domains form biomolecular condensates
Source: J Cell Commun Signal. 2025 Jul 26;19(3):e70039. doi: 10.1002/ccs3.70039 (PMC13674738; doi:10.1002/ccs3.70039)
Supplement: Supplementary file 2 [file CCS3-19-e70039-s002.docx]

Supplemental table 1:

| **Plasmid #**  **Plasmid name** | **Desired Construct/ Primer Function** | **Amino Acids** | **Dir.** | **Sequence (5'→3')** | |
| --- | --- | --- | --- | --- | --- |
| 420  Flag-N1ICD-Δ70 | Serial truncations of N1ICD C-terminal domain | V1744  -  S2461 | FWD | TAA GGA TCC CGG GTG GCA TCC CTG T | |
|  |  |  | REV | CAC CCG GGA TCC TTA GGA AGG AGG GGT CAG GA | |
| 421  Flag-N1ICD-Δ140 |  | V1744  -Q2391 | FWD | TAA GGA TCC CGG GTG GCA TCC CTG T | |
|  |  |  | REV | CAC CCG GGA TCC TTA CTG TGA TGG TGG CTG CA | |
| 422  Flag-N1ICD-Δ210 |  | V1744  -  T2321 | FWD | TAA GGA TCC CGG GTG GCA TCC CTG T | |
|  |  |  | REV | CAC CCG GGA TCC TTA CGT CAC ACC CGG CC | |
| 423  Flag-N1ICD- Δ280 |  | V1744  -  G2251 | FWD | TAA GGA TCC CGG GTG GCA TCC CTG T | |
|  |  |  | REV | CAC CCG GGA TCC TTA ACC TCC AGC CAG TGC T | |
| 424  Flag-N1ICD- Δ350 |  | V1744  -  L2181 | FWD | TAA GGA TCC CGG GTG GCA TCC CTG T | |
|  |  |  | REV | CAC CCG GGA TCC TTA CAA CAG GCA GCC CTT G | |
| 380  Flag-N1ICD- ΔCTD |  | V1744 | FWD | TAA GGA TCC CGG GTG GCA TC | |
|  |  |  | REV | GCT GCG CAC CAG GTT GTA C | |
|  |  |  | REV | GGC TTC CGG TGC CTA CCC GCG CGG GCG | |
| 436  Flag-N1ICDΔ2111-2181 | Deletion of 70 amino acids directly C-term of the Ank domain | P2112 –  L2181 | FWD | GAC AGC TCG AGC ATG CTG | |
|  |  |  | REV | GCTGCGCACCAGGTTGTA | |
| 444  Flag-N3ICD+N1 2111-2181 | Addition of amino acids from N1ICD to C-term of the Ank domain | P2032 –  L2101 | Insert | FWD | CAGTGGACCTCCACAGCTGCATGGCACTG |
|  |  |  |  | REV | AGGGACTTCGCAACAGGCAGCCCTTGCC |
|  |  |  | Vector | FWD | CTGCCTGTTGCGAAGTCCCTCTGGTCCCC |
|  |  |  |  | REV | GCAGCTGTGGAGGTCCACTGGGCTGGTC |
