# Supplementary material for: Notch NICD domains form biomolecular condensates
Source: J Cell Commun Signal. 2025 Jul 26;19(3):e70039. doi: 10.1002/ccs3.70039 (PMC13674738; doi:10.1002/ccs3.70039)
Supplement: Supplementary file 3 [file CCS3-19-e70039-s004.docx]

Supplementary Figure and Table Legends:

Supplementary Figure 1: AlphaFold3 was used to predict IDR domains in N1ICD. Shown are the top five AlphaFold structures overlayed. Top panel highlights each of the five structures and bottom panel shows plDDT scores for each of five structures.

Supplementary Figure 2A: ClustalW alignment of NICD domains from hagfish (Myxine glutinosa), human (Homo sapiens), fly (Drosophila melanogaster), and sponge (Amphimedon queenslandica). Ankyrin domains are indicated in shaded sequence and are based on human ankyrin domains as defined by Lubman et al., 2004 [63].

Supplementary Figure 2B: Species comparison of NICD IDRs. IDR predictions were made by flDPnn in the NICD domains of mouse (Mus musculus), hagfish (Myxine glutinosa), human (Homo sapiens), fly (Drosophila melanogaster), and sponge (Amphimedon queenslandica). The position of each ankyrin domain is indicated by the box on each graph. Ankyrin domains were estimated based on sequence alignment with human ankyrin domain as described in Lubman et al., 2004 [63]. The red line in each figure indicates the default threshold where sequences above the red line are predicted to be disordered.

Supplementary table 1: Plasmids constructed. Plasmids constructed for this study, specific mutation sites, and oligos used to create mutations by PCR (plasmids #429, 421, 422,423,424, 380, and 436) or Gibson assembly (plasmid #444) are indicated.

Supplementary table 2: Antibodies used. Primary and secondary antibodies, working dilutions / concentrations, vendor, and product numbers are indicated.
